# *Drosophila* SETDB1 Is Required for Chromosome 4 Silencing 

**DOI:** 10.1371/journal.pgen.0030076

**Published:** 2007-05-11

**Authors:** Carole Seum, Emanuela Reo, Hongzhuang Peng, Frank J Rauscher, Pierre Spierer, Séverine Bontron

**Affiliations:** 1 Department of Zoology and Animal Biology, University of Geneva, Geneva, Switzerland; 2 The Wistar Institute, Philadelphia, Pennsylvania, United States of America; European Molecular Biology Laboratory, Germany

## Abstract

Histone H3 lysine 9 (H3K9) methylation is associated with gene repression and heterochromatin formation. In *Drosophila*, SU(VAR)3–9 is responsible for H3K9 methylation mainly at pericentric heterochromatin. However, the histone methyltransferases responsible for H3K9 methylation at euchromatic sites, telomeres, and at the peculiar Chromosome 4 have not yet been identified. Here, we show that DmSETDB1 is involved in nonpericentric H3K9 methylation. Analysis of two *DmSetdb1* alleles generated by homologous recombination, a deletion, and an allele where the 3HA tag is fused to the endogenous *DmSetdb1,* reveals that this gene is essential for fly viability and that DmSETDB1 localizes mainly at Chromosome 4. It also shows that DmSETDB1 is responsible for some of the H3K9 mono- and dimethyl marks in euchromatin and for H3K9 dimethylation on Chromosome 4. Moreover, DmSETDB1 is required for variegated repression of transgenes inserted on Chromosome 4. This study defines DmSETDB1 as a H3K9 methyltransferase that specifically targets euchromatin and the autosomal Chromosome 4 and shows that it is an essential factor for Chromosome 4 silencing.

## Author Summary

DNA is the basic unit carrying genetic information. Within the nucleus, DNA is wrapped around an eight-histone complex to form the nucleosome. The nucleosomes and other associated proteins assemble to a higher order structure called chromatin. The histones are mainly globular, excepted for their tails that protrude from the nucleosome core. The amino acids of the histone tails are often modified. For example, several conserved lysine residues can be methylated. Methylation of lysine 9 on histone H3 (H3K9) is important for proper chromatin structure and gene regulation. Here, we characterize *Drosophila* DmSETDB1 as a histone methyltransferase responsible for H3K9 methylation of the chromosome arms and Chromosome 4. In addition, we show that in the absence of DmSETDB1, silencing of Chromosome 4 is abolished. This study is an important step towards the understanding of the differential chromatin domain specificity and mode of action of H3K9 methyltransferases.

## Introduction

Methylation of conserved lysine residues on histone H3 and H4 tails plays a key role in gene regulation, chromatin structure, and establishment and maintenance of epigenetic memory (reviewed in [1]). As proposed by the “histone code” hypothesis [2], these marks, in association with other modifications, are interpreted by chromatin-specific regulatory complexes that in turn influence chromatin structure and its accessibility to transcription factors. Euchromatin is characterized by histone H3 methylated at lysine 4 (K4), K36, and K79, while heterochromatin is characterized by histone H3 methylated at K9 and K27 and histone H4 methylated at K20 [1]. Moreover, histone methylation can be present in mono-, di-, or trimethylation states [3,4]. All but one enzyme responsible for histone lysine methylation share an evolutionary conserved domain of about 130 amino acids, called the SET domain [5,6]. Numerous SET domain-containing proteins responsible for methylation of specific residues have been described in all eukaryotic organisms (reviewed in [7]). Enzymes with histone demethylase activity were only recently characterized [8].

In *Drosophila,* similarly as in other organisms, histone H3 lysine 9 (H3K9) methylation plays a crucial role for heterochromatin formation and maintenance and for gene silencing. Methylated H3K9 is a docking site for the recruitment of the heterochromatin protein 1 (HP1) through its chromodomain [9–11]. *Su(var)3–9* was the first H3K9 methyltransferase characterized in *Drosophila* [12]. It was historically identified in genetic screens, together with *Su(var)2–5* encoding HP1 and *Su(var)3–7*, as a haplo-suppressor and triplo-enhancer of position effect variegation [13], a phenomenon that reflects the mosaic heterochromatin-induced silencing of genes. SU(VAR)3–9 is responsible for H3K9 dimethylation at the chromocenter and trimethylation at the core of the chromocenter, but not for H3K9 monomethyl marks at the chromocenter and along the chromosome arms, nor for the dimethyl marks at the chromosome arms, telomeres, and Chromosome 4 [14,15]. Recently, *Drosophila* dG9a was shown to display H3K9- as well as H3K27- and H4-methyltransferase activity, to localize at discrete bands in euchromatin, and to be excluded from Chromosome 4 [16,17], suggesting that it methylates H3K9 at euchromatic sites. But the histone methyltransferases (HMTases) that methylate H3K9 outside the chromocenter have not been formally characterized.

Drosophila melanogaster's Chromosome 4 is the smallest autosome and displays a peculiar chromatin organization (for a review on Chromosome 4 see [18]). It is mostly heterochromatic, composed of a highly condensed ~3–4-Mb centromeric region that is under-replicated and a 1.2-Mb polytenized arm exhibiting a banded pattern. The banded region displays characteristics typical of heterochromatin based on a number of criteria: transposable and repetitive elements are represented at a high density [19,20], P elements often display a variegated expression [21–23], H3K9 dimethyl marks are present [14,15], and HP1 is distributed in a banded pattern [24]. Surprisingly, in opposition to these heterochromatic characteristics, the banded portion shows a gene density comparable to euchromatin; many of these genes are essential, therefore expressed during development [25,26]. In addition, the H3K9 dimethyl mark is not deposited by the heterochromatic SU(VAR)3–9 [14,15]. These features converge to the conclusion that chromatin of the Chromosome 4 banded region is different from centromeric heterochromatin.

Human SETDB1 (mouse ESET) is an essential H3K9 methyltransferase involved in silencing in euchromatin [27–30]. It is composed of a Tudor-, a methyl CpG binding- (MBD), and a bifurcated SET-domain that is surrounded by pre- and post-SET domains [31]. Recently, the D. melanogaster homologue gene of SETDB1 (named *dsetb1, eggless,* or *dEset*) was identified; the domains characteristic of mammalian *SETDB1* are well conserved, reaching 76% identity in the SET-C terminus and post-SET domains [16,32,33]. In addition, an histone deacetylase-interacting domain was identified [33]. This gene was shown to be involved in H3K9 trimethylation both in germ and somatic cells of the germarium and to be required for oogenesis at early stages of egg chamber formation [32].

Here, we show that DmSETDB1 is the missing euchromatin- and Chromosome 4-specific H3K9 HMTase. We generated a *DmSetdb1* mutant allele and a 3HA-tagged *DmSetdb1* allele by homologous recombination and show that this gene is essential for fly viability and that the endogenous DmSETDB1 protein localizes mainly at Chromosome 4. In addition, we evidence that DmSETDB1 is responsible for some H3K9 mono- and dimethyl marks in euchromatin, as well as for Chromosome 4 H3K9 dimethylation. Moreover, DmSETDB1 turned out to be required for repression of variegating transgenes inserted on Chromosome 4, a function that is consistent with the role of DmSETDB1 in Chromosome 4 H3K9 dimethylation. Therefore, DmSETDB1 is a key H3K9 methyltransferase in *Drosophila* involved in repression of the peculiar Chromosome 4.

## Results

### *DmSetdb1* Is an Essential Gene Expressed throughout Development

**Figure 1:**
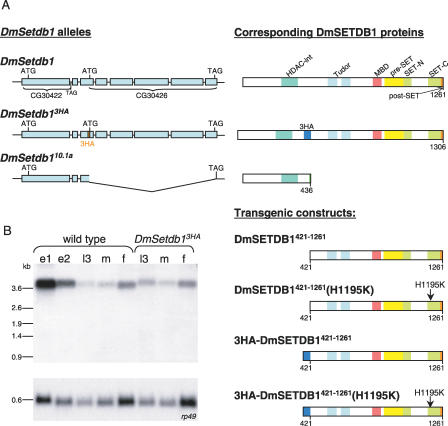
Schematic of the *DmSetdb1* Alleles and Expression Profile of *DmSetdb1* during Development (A) The structure of the *DmSetdb1* gene is represented as well as the two alleles generated by homologous recombination. The gene was previously annotated as two entities, *CG30422* and *CG30426*. *DmSetdb1^3HA^* consists in a 3HA tag inserted in phase in the third exon. *DmSetdb1^10.1a^* is a deletion removing amino acids 421 to 1,261 of the protein. The protein products of the three *DmSetdb1* alleles and of transgenic constructs used in this study are also represented. (B) Analysis of *DmSetdb1* expression by Northern blot in wild-type and *DmSetdb1^3HA^* homozygous background at the indicated developmental stages: e1, 0–4-h embryos; e2, 0–18-h embryos; l3, third instar larvae; m, adult males; f, adult females. Membranes were hybridized with a probe spanning nucleotides 2,399 to 3,789 of *DmSetdb1* ORF and with a RNA loading control probe recognizing the 0.6-kb *rp49* transcript.

The open reading frame (ORF) of *CG30426* was identified by protein BLAST search (National Center for Biotechnology Information [NCBI], http://www.ncbi.nlm.nih.gov) as the closest *Dm* homologue of the human H3K9 methyltransferase *SETDB1*. Others also identified *CG30426* by protein BLAST or in a SET-domain phylogenic tree as the *Dm* homologue of *SETDB1* [16,34]. Subsequently, *CG30426* and the neighboring *CG30422* (see representation Figure 1A) were shown to produce a single 3.9-kb mRNA transcript in ovaries [32] and constitute a single transcript in females [33], suggesting that the *DmSetdb1* gene is composed of both *CG30422* and *CG30426*. As a full insert cDNA corresponding to *CG30426* alone (AT13877) is present in public databases, we addressed whether the *DmSetdb1* gene was transcribed from several transcription start sites, subjected to alternative splicing in a tissue-specific manner, or if the 3.9-kb transcript was the unique product. Northern blot analysis shows that in embryos, third instar larvae, male and female adults, a single 3.9-kb transcript is detected with a probe specific for *CG30426,* with a stronger signal in embryos (Figure 1B). The same profile is obtained with a probe spanning *CG30422* (unpublished data). Therefore, *DmSetdb1* is expressed as a 3.9-kb transcript encompassing both *CG30422* and *CG30426,* which is present at all developmental stages and encodes a 1,261-amino acid protein.

To study DmSETDB1 function in vivo, we generated the *DmSetdb1^10.1a^* mutant allele by homologous recombination [35,36]. In this allele, amino acids 421 to 1,261 comprising the Tudor, MBD, pre-SET, SET-N, SET-C, and post-SET domains are deleted. The entire ORF was not removed because when the present study was designed, *CG30422* was not considered part of the *DmSetdb1* gene. The 5′-end of the *DmSetdb1* gene is transcribed in the *DmSetdb1^10.1a^* allele (unpublished data), therefore the 420 first amino acids of DmSETDB1 are potentially translated, followed by 16 unrelated amino acids and a stop codon (Figure 1A). This mutation is recessive lethal, in that homozygotes die at late pupal stage, with no escapers. The same phenotype is observed in individuals transheterozygous for *DmSetdb1^10.1a^* and the chromosomal deficiency Df(2R)ED4065 deleting the *DmSetdb1* gene (deleted segment: 60C8-60E7). The polytene chromosomes of homozygote *DmSetdb1^10.1a^* larvae appear normal (Figure S1). The *DmSetdb1^10.1a^* homozygous mutant flies can be rescued into the adult stage by expression of DmSETDB1^421–1,261^ or 3HA-DmSETDB1^421–1,261^ transgenes (*UAS- DmSetdb1^421^* ^–*1,261*^ *daGal4* heterozygotes). The rescued females are sterile, while the males are fertile, leading to the conclusion that DmSETDB1^421–1,261^ is partially functional. Collectively, phenotypic analysis of *DmSetdb1^10.1a^* homozygotes and of transheterozygotes for *DmSetdb1^10.1a^* and the chromosomal deficiency Df(2R)ED4065 shows that *DmSetdb1* is an essential gene, and that *DmSetdb1^10.1a^* behaves as a null allele.

### DmSETDB1 Localizes at Chromosome 4, Euchromatin and Chromocenter, and Is Involved in Some H3K9 Mono- and Dimethyl Marks in Euchromatin and in H3K9 Dimethylation of Chromosome 4

**Figure 2:**
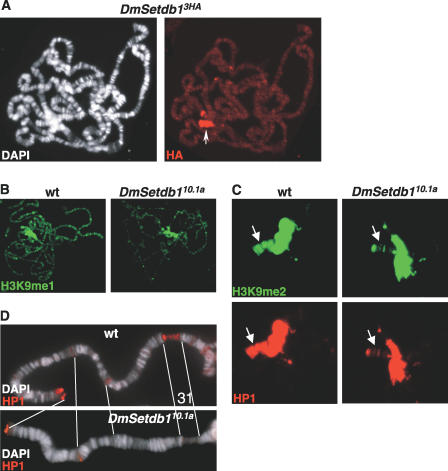
Localization of Endogenous DmSETDB1 and Comparison of H3K9me1, me2, and HP1 Pattern between Wild-Type and *DmSetdb1^10.1a^* Homozygote Mutant (A) Salivary gland polytene chromosomes of *DmSetdb1^3HA^* homozygous larvae were stained with α-HA. DNA is stained with 4′,6-diamidino-2-phenylindole (DAPI). DmSETDB1 localizes at Chromosome 4 (white arrows) and euchromatic arms. The chromocenter is also stained. (B) Salivary gland polytene chromosomes of wild-type (wt) and *DmSetdb1^10.1a^* homozygous mutant (*DmSetdb1^10.1a^*) were immunostained with α-H3K9me1. In the mutant, the H3K9me1 signal persists at the chromocenter but is weaker on the euchromatic arms when compared to wild type. (C) Immunostaining with antibodies recognizing H3K9me2 or HP1. Pictures were taken at higher magnification. White arrows show the Chromosome 4 arm. In the *DmSetdb1^10.1a^* homozygous mutant H3K9me2 and HP1, signals are lost on most of Chromosome 4 arm but not at chromocenter or telomere. (D) Immunostaining with a α-HP1 is shown. DNA is stained with DAPI. Pictures were taken at higher magnification and show chromosome 2R arm. The region 31 is bound by HP1 in wild-type condition but not in *DmSetdb1^10.1a^* homozygous mutant. Telomere is bound by HP1 in both genetic backgrounds.

We next investigated the biological function of endogenous DmSETDB1. We first looked at the localization of the endogenous DmSETDB1 on polytene chromosomes. Therefore we generated the *DmSetdb1^3HA^* allele by homologous recombination, which produces the endogenous DmSETDB1 protein tagged internally with a 3HA (Figure 1A). *DmSetdb1^3HA^* is expressed at a similar level compared with the wild-type allele (Figure 1B). The transcript of *DmSetdb1^3HA^* is slightly longer than that of the wild-type allele due to the 3HA sequence (Figure 1B); it was amplified by reverse transcriptase-PCR and sequenced, and shows no aberrant splicing (unpublished data). *DmSetdb1^3HA^* homozygous flies are viable and can be maintained as a stock, showing that the 3HA tag does not impair DmSETB1 function. Staining of homozygous *DmSetdb1^3HA^* larvae polytene chromosomes with anti-HA shows a strong signal on Chromosome 4 (Figure 2A). DmSETDB1 is also present over the whole length of the euchromatic arms, with some spots being more occupied. The chromocenter is weakly stained (Figure 2A), a feature whose significance needs to be studied further, as DmSETDB1 is not methylating the chromocenter (see below). As a negative control, polytene chromosomes of wild-type larvae stained with anti-HA show no signal (unpublished data). Thus, endogenous DmSETDB1 localizes at Chromosome 4 and chromosome arms.

**Figure 3:**
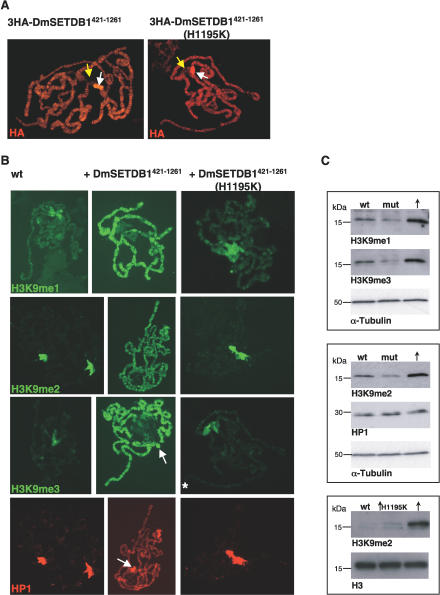
Overexpression of DmSETDB1^421–1,261^ Induces Increase in H3K9 Mono-, Di-, and Trimethylation (A) Salivary glands polytene chromosomes from larvae expressing 3HA-DmSETDB1^421–1,261^ (*CaSpeR-3HA-DmSetdb1^421–1,261^*) or 3HA-DmSETDB1^421–1,261^(H1195K) (*UAS-3HA- DmSetdb1^421–1,261^(H1195K) daGal4* homozygotes) were immunostained with α-HA. For the 3HA-DmSETDB1^421–1,261^, larvae were subjected 30 min at 37 °C heat shock and placed 1 h at room temperature for recovery before squashing. 3HA-DmSETDB1^421–1,261^ and 3HA-DmSETDB1^421–1,261^ (H1195K) localize at the Chromosome 4 and on the euchromatic arms. White arrows point at the Chromosome 4 and yellow arrows at the chromocenter. (B) Immunostaining of salivary gland polytene chromosomes of wild-type (wt), overexpressing DmSETDB1^421–1,261^ (*UAS-DmSetdb1^421–1,261^ daGal4* homozygotes), and overexpressing mutant DmSETDB1^421–1,261^(H1195K) (*UAS-DmSetdb1^421–1,261^(H1195K) daGal4* homozygotes) third instar larvae. The chromosome with a white asterisk corresponds to the overexpression of the 3HA-tagged protein. Chromosomes were stained with antibodies recognizing H3K9me1, me2, me3, or HP1. Overexpression of DmSETDB1^421–1,261^ but not of mutant DmSETDB1^421–1,261^(H1195K) leads to a strong increase of H3K9me1, me2, and me3 signals on the euchromatic arms and on the Chromosome 4 (white arrow on H3K9me3 panel); moreover HP1 is recruited to euchromatic arms and strongly binds Chromosome 4 (white arrow on HP1 panel). (C) Top and middle panels: Western Blot analysis on 20 μg brain, salivary glands, and imaginal discs extracts (dissected together) of wild-type (wt), *DmSetdb1^10^* ^.1a^ homozygote mutant (mut), overexpressing DmSETDB1^421–1,261^ (↑) (*UAS-DmSetdb1^421–1,261^ daGal4* heterozygotes) third instar larvae. First membrane was probed with α-H3K9me1 and α-α-tubulin, stripped, and probed with α-H3K9me3. Second membrane was probed with α-H3K9me3 and α-tubulin, stripped, and probed with α-HP1. Bottom panel: 5 μg brain extracts from wild-type (wt), overexpressing mutant DmSETDB1^421–1,261^(H1195K) (↑H1195K) (*UAS- DmSetdb1^421–1,261^[H1195K] daGal4* homozygotes), and overexpressing DmSETDB1^421–1,261^ (↑) (*UAS-DmSetdb1^421–1,261^ daGal4* homozygotes) of third instar larvae were loaded twice and membranes were probed with α-H3K9me2 or α-H3.

By analogy to mammalian SETDB1, which is a H3K9 mono-, di-, and tri-HMTase [27,28], we asked whether DmSETDB1 is responsible for some of the H3K9 methyl marks present in chromatin. To address this, H3K9 mono-, di-, and trimethyl marks of wild-type and homozygous *DmSetdb1^10.1a^* mutant larvae on polytene chromosomes were compared. Similarly as described in the literature, in wild-type conditions, the H3K9 monomethyl antibody stains the chromocenter and the euchromatic arms, although faintly. In the *DmSetdb1^10.1a^* mutant, the monomethyl H3K9 signal is less intense on euchromatin, but does not completely disappear. However, the signal at the chromocenter remains unchanged (Figure 2B). Therefore, DmSETDB1 is involved in some but not in all of the euchromatic H3K9 monomethylation, and displays no activity at the chromocenter. The H3K9 dimethyl antibody stains the chromocenter and Chromosome 4 in wild-type larvae, while the telomeres and the few euchromatic bands that were shown to bear H3K9 dimethyl marks [14,15] are not easily detectable. In the *DmSetdb1^10.1a^* mutant background the mark is strongly reduced at the arm of Chromosome 4, while the telomere and chromocenter are not affected (Figure 2C). As a consequence, HP1 is present at the chromocenter and at the telomere, but it is not recruited to the Chromosome 4 arm, except for a few signals visualized as faint bands (Figure 3B). Loss of HP1 at Chromosome 4 reinforces the conclusions made with the H3K9 dimethyl staining, namely that DmSETDB1 is the H3K9 dimethyl HMTase of the Chromosome 4 arm. We wanted to analyze the euchromatic and telomeric H3K9 dimethyl marks of the other chromosomes in *DmSetdb1* mutant larvae, but the currently available antibodies do not allow detection of these marks. To circumvent this technical problem, stainings were performed with an antibody recognizing HP1 that produces significant signals. In the *DmSetdb1^10.1a^* mutant background, HP1 is present on telomere, but disappears from some bands known to be strongly enriched in H3K9 dimethyl and HP1, as for instance region 31 of Chromosome 2 [37] (Figure 2D). Telomeres of the other chromosome arms are also bound by HP1 in the *DmSetdb1^10.1a^* mutant background. Taken together, these results show that DmSETDB1 has an H3K9 dimethyl HMTase activity at some sites on the euchromatic arms, at Chromosome 4, but not at telomeres.

In terms of the H3K9 trimethyl modification present at the core of the chromocenter and few sites on the chromosome arms, we could not detect any difference between wild-type and *DmSetdb1^10.1a^* homozygous mutant polytene chromosomes (unpublished data). We also examined other methylation marks associated with repression, namely mono- and dimethyl H3K27, and could not show any change in the *DmSetdb1^10.1a^* mutant background (unpublished data), arguing in favor of the specificity of DmSETDB1 for H3K9. From these data we conclude that DmSETDB1 is responsible for some of the H3K9 mono- and dimethyl marks in euchromatin, and for most of Chromosome 4 H3K9 dimethylation. Others HMTases must be responsible for persistent H3K9 mono- and dimethylation in euchromatin, for H3K9 monomethylation at the chromocenter, and for H3K9 dimethylation at the telomeres.

### Overexpression of DmSETDB1^421–1,261^ Is Lethal and Leads to Increased H3K9 Methylation and to the Recruitment of HP1

We next asked if overexpression of DmSETDB1 induces an increase of H3K9 methylation. This would confirm the ability of DmSETDB1 to mono- and dimethylate H3K9 and address if it can trimethylate H3K9, as described for its mammalian homologue [27,28]. In addition, this would show whether DmSETDB1 is a limiting factor for the H3K9 methylation level or not. We overexpressed DmSETDB1^421–1,261^, a less than full-length protein that nonetheless contains the Tudor, MBD, pre-SET, SET, and post-SET domains and can rescue the *DmSetdb1^10.1a^* homozygotes (see above). In addition, the 3HA-tagged version of DmSETDB1^421–1,261^ localizes similarly to the full-length protein, namely at Chromosome 4 and at euchromatin, although the signal is stronger at euchromatin most likely because of its higher expression (Figure 3A). It is not possible to assess whether H3K9 methylation is present at the chromocenter, since it becomes disorganized upon DmSETDB1 overexpression (Figure S1). Thus, we consider that DmSETDB1^421–126^ is suitable to study the HMTase activity of DmSETDB1. Increased expression of *DmSETDB1^421–1,261^* is lethal, as ubiquitously overexpressing flies *(UAS-DmSetdb1^421–1,261^ daGal4* homozygotes) die during the pupal stage, while heterozygous individuals survive and are fertile. Polytene chromosomes of larvae overexpressing DmSETDB1^421–1,261^ show an aberrant morphology. They appear thickened with unusual constrictions, and the chromocenter looks disorganized and decondensed (Figure S1). Such chromatin defects could be the cause of lethality. Upon DmSETDB1^421–1,261^ overexpression, there is a strong increase in H3K9 mono-, di-, and trimethylation on all chromosome arms, including Chromosome 4 (Figure 3B). As a control, H3K27 mono- and dimethyl marks do not change when DmSETDB1 is overexpressed (unpublished data). The same stainings were repeated under conditions where the DmSETDB1^421–1,261^(H1195K) protein is overexpressed. The histidine 1,195 position is invariant among the SET proteins and is part of the cofactor AdoMet-binding pocket. The corresponding point mutation in human *SETDB1* abolishes HMTase activity [27]. 3HA-DmSETDB1^421–1,261^(H1195K) localizes similarly as 3HA-DmSETDB1^421–1,261^ (Figure 3A), showing that the enzymatic activity is not required for chromatin localization of DmSETDB1^421–1,261^. Overexpression of the mutant protein does not induce any increase or change in the H3K9 mono-, di-, or trimethylation patterns (Figure 3B).

HP1 recognizes H3K9 di- and trimethylated histones [9–11] and localizes at the chromocenter, the telomeres, Chromosome 4, and at approximately 200 euchromatic sites of wild-type polytene chromosomes [24]. We wondered whether the profile of HP1 would be altered under DmSETDB1-overexpressing conditions. When DmSETDB1^421–1,261^ is overexpressed, HP1 is absent from the loose chromocenter, remains on Chromosome 4, and is recruited to the euchromatic arms, more intensely at some sites (Figure 2B). Western blot analysis shows that the total amount of HP1 is similar in DmSETDB1^421–1,261^ overexpressing and in wild-type larvae (Figure 3C). These results indicate that HP1 is not expressed in larger amounts nor stabilized. Recruitment of HP1 to the chromosome arms does not occur upon overexpression of the DmSETDB1^421–1,261^(H1195K) mutated protein (Figure 2B), showing that DmSETDB1^421–1,261^ alone cannot recruit HP1. Taken together, these results show that overexpressed DmSETDB1^421–1,261^ is located at and has an H3K9 mono-, di-, and tri-HMTase activity on the euchromatic arms and on Chromosome 4, leading to the recruitment of HP1.

Global levels of H3K9 mono-, di-, and trimethylation were also measured by western blot analysis in tissue extracts from wild type, overexpressing DmSETDB1^421–1,261^ and *DmSetdb1^10.1a^* homozygote mutant third instar larvae. Overexpression of DmSETDB1^421–1,261^ markedly increases mono-, di-, and trimethyl H3K9 levels, whereas absence of DmSETDB1 results in a modest decrease of these three modifications (Figure 3C, first and second panels). The reduction observed in the *DmSetdb1^10.1a^* mutant background is subtle but reproducible. Total H3 and HP1 levels (unpublished data and Figure 3C, second panel) are not influenced by the overexpression or the absence of DmSETDB1. As expected, overexpression of the DmSETDB1^421–1,261^(H1195K) mutant protein has no effect on H3K9 dimethylation (Figure 3C, third panel) or trimethylation (unpublished data) levels, except for a subtle increase in signal strength. Note that the increase in H3K9 dimethylation upon DmSETDB1^421–1,261^ overexpression is stronger in the third compared to the second panel, because the larvae are homozygous for the transgene. We conclude that DmSETDB1 is an H3K9 mono-, di-, and tri-HMTase and that increased expression positively influences the H3K9 methylation level.

### DmSETDB1 Is Required for Repression of Variegating Transgenes Inserted on Chromosome 4

**Figure 4:**
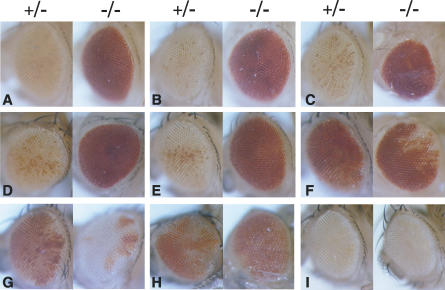
Chromosome 4 Variegating Transgenes Are Derepressed in *DmSetdb1^10.1a^* Homozygotes Eyes pictures showing expression of variegating *white* transgenes on Chromosome 4 (A–E) and other chromosomes (F–I), in *DmSetdb1^10.1a^* heterozygous (+/−) and homozygous (−/−) background. Wild-type flies show the same phenotype as *DmSetdb1^10.1a^* heterozygotes (unpublished data). Pictures show heterozygous and not wild-type flies, because heterozygous and homozygous mutant flies were generated from the same cross and are thus directly comparable. Genotypes were analyzed by PCR. (A) corresponds to the *39C12* P element, (B) to *39C72*, (C) to *118E10*, (D) to *6M193*, (E) to *118E15*, (F) to *39C3*, (G) to the *In(1)w^m4h^* inversion, (H) to *Heidi,* and (I) to *39C5*. P elements in (A–E) are inserted on Chromosome 4 arm, in (F) and (H) near Chromosome 2 centromeric heterochromatin, in (I) near telomere of 2L, and (G) is an inversion on Chromosome X relocating the endogenous *white* gene next to centromeric heterochromatin. For genotypes, see Materials and Methods.

Given that DmSETDB1 strongly localizes to and methylates H3K9 on Chromosome 4, we next assessed its role in gene regulation on that peculiar chromosome. To do this, we analyzed whether DmSETDB1 level would affect expression of *white* transgenes when placed on Chromosome 4. Therefore, we used previously characterized lines where the *white* gene is expressed from P elements inserted in or at the edge of Chromosome 4 heterochromatic interspersed domains [21,22,38–40]. These lines display a variegated phenotype, indicating that the *white* gene is stochastically silenced. This pattern is reminiscent of heterochromatic position-effect variegation on other chromosomes, and mutations in *HP1* or *Su(var)3–7* result in re-expression of the *white* gene, in all but one line (39C5) [21,38–40]. On the other hand, these variegating reporters do not respond to an additional or missing dose of SU(VAR)3–9 (mentioned in [18] as personal communication from K. Haynes [41]). If DmSETDB1 were implicated in repression via its HMTase activity, its absence would lead to reactivation of *white* expression. In parallel, four variegating lines were tested, two that have P elements inserted near centromeric heterochromatin of Chromosome 2, one that has a P element inserted in the subtelomeric region of 2L, and the other being the *In(1)w^m4h^* line, in which an inversion relocates the endogenous *white* gene next to centromeric heterochromatin. *White* expression was analyzed in wild-type *DmSetdb1^10.1a^* heterozygous and homozygous mutant late pupae. In the heterozygous mutant background, none of these lines differs from the wild type (unpublished data). In the *DmSetdb1^10.1a^* homozygous mutant context, however, the lines with transgene on Chromosome 4 show a robust expression of the *white* reporter (Figure 4, compare *DmSetdb1* +/− and −/− in panels A–E). This is neither the case for the three transgenes on Chromosome 2 (H, F, and I), nor for the *white* gene on the X Chromosome (G). The expression in the *In(1)w^m4h^* line (G) is even reproducibly lower in the absence of DmSETDB1, for as yet not understood reasons. These results show that *DmSetdb1* is a recessive suppressor of variegation of Chromosome 4. In the absence of DmSETDB1, repression of transgenes located in the vicinity of Chromosome 4 heterochromatic domains is abolished.

## Discussion

In D. melanogaster, the enzyme(s) responsible for H3K9 methylation at euchromatin, telomeres, and at the peculiar autosomal Chromosome 4 have not yet been characterized. Here, we identify DmSETDB1 as a major H3K9 methyltransferase at euchromatin and Chromosome 4. We demonstrate that *DmSetdb1* is an essential gene, and that DmSETDB1 is required for Chromosome 4 silencing. Thus, DmSETDB1 is the second H3K9 methyltransferase characterized in *Drosophila,* the first one being the heterochromatin-specific SU(VAR)3–9.

### *DmSetdb1* Is an Essential Gene

Whereas *Su(var)3–9* and *dG9a* are not essential ([42], C. Seum, unpublished data), *DmSetdb1* is the first gene described encoding a H3K9 methyltransferase that is required for fly viability. *DmSetdb1* transcript can be detected at every stage of development. Our analysis by Northern blot confirms that the only transcript is 3.9 kb long, encompassing both CG30422 and CG30426. Early embryos show relative high mRNA levels, suggesting deposition of the transcript in the embryo. Others conclude that *DmSetdb1* transcript is not present in 0–3-h embryos when tested by reverse transcriptase-PCR [33], a result that is not easily reconciled with our observations. *DmSetdb1^10.1a^* homozygotes are rescued with the *UAS- DmSetdb1^421–1,261^ daGal4* transgene; the males are fertile, while the females are sterile. Thus, the rescue is not complete in females, because of either nonappropriate expression of the transgene or because DmSETDB1^421–1,261^ is not full-length. This observation is consistent with the fact that *DmSetdb1 (eggless)* was shown to be required for oogenesis [32]. Preliminary data suggest that sterility in rescued females and in *eggless* mutant alleles [32] is due, at least in part, to defects in germline development. Indeed, using the FLP-ovo^D1^ system [43], we could not generate any *DmSetdb1^10.1a^* homozygous mutant germline clone (unpublished data). This suggests that germline-specific expression of *DmSetdb1* is required before stage 5 of oogenesis. This does not exclude, however, that a maternal contribution is required for proper oogenesis.

### DmSETDB1 Localizes at Chromosome 4 and Euchromatin

The polyclonal antibody directed against a DmSETDB1 peptide we generated does not recognize DmSETDB1 on polytene chromosomes. Therefore, we generated the *DmSetdb1^3HA^* allele that results into the expression of the endogenous DmSETDB1 protein tagged with 3HA (Figure 1A). Such an approach has the advantage that the endogenously expressed protein can be detected with highly specific monocolonal antibodies. This allowed us to show that DmSETDB1 localizes at a high level on Chromosome 4 and over the chromosome arms (Figure 2A). DmSETDB1 is also present at the chromocenter. We do not know if this feature has any biological significance as DmSETDB1 does not methylate the chromocenter. The association of DmSETDB1 with chromatin is not dependent on its own catalytic activity, since the DmSETDB1^421–1,261^(H1195K) mutant protein localizes similarly to DmSETDB1^421–1,261^ (Figure 3A). The mode of DmSETDB1 recruitment thus differs from that of SU(VAR)3–9, since the latter appears to require its HMTase activity for binding to heterochromatin [44]. It is currently not known how DmSETDB1 is recruited to chromatin. Mammalian SETDB1 is recruited to DNA together with HP1, either via the KRAB-zinc-finger protein KAP1 corepressor [27,45] or by the ERG transcription factor [46], or as a component of the MBD1-mAM/MCAF1-SETDB1 complex [30,47,48]. It is tempting to speculate that in *Drosophila* transcriptional repressors also recruit DmSETDB1 onto euchromatin or at Chromosome 4.

### DmSETDB1 Is a Euchromatin- and Chromosome 4-H3K9 Methyltransferase

Comparative analysis of H3K9 methylation and HP1 profile on polytene chromosomes of wild-type and *DmSetdb1^10.1a^* homozygous mutant larvae shows that DmSETDB1 is involved in some of the H3K9 mono- and dimethyl marks in euchromatin and in dimethyl marks on Chromosome 4 (Figure 2B and 2C). Loss of methylation at Chromosome 4 and euchromatin is coherent with the localization profile of the DmSETDB1 protein itself. Western blot analysis of the H3K9 methylation level in mixed salivary glands, brain, and imaginal discs tissue in *DmSetdb1* mutant background shows a decrease in all three H3K9 methyl marks (Figure 3C). We could not evidence any change of trimethylation in polytene chromosomes of *DmSetdb1^10.1a^* mutant larvae. This suggests a distinct H3K9 trimethylation profile in the tissues analyzed by Western blot and in polytene chromosomes. This hypothesis is corroborated by the recent finding that DmSETDB1 trimethylates H3K9 in germ and somatic cells of the germarium [32].

The overexpression data provide a mirror image, in that they show the ability of DmSETDB1 to mono-, di-, and trimethylate H3K9 (Figure 3). Thus, *Drosophila* DmSETDB1 and mammalian SETDB1 are conserved with respect to their HMTase activity, as both *Drosophila* DmSETDB1 and mammalian SETDB1 are H3K9 mono-, di-, and tri-HMTases [27,28]. Although such a mechanism has not yet been described, we cannot exclude that DmSETDB1 is exclusively a H3K9 monomethyltransferase providing monomethyl substrates for other enzymes; but in that case, the partner enzyme would not be SU(VAR)3–9, since its absence does not impair Chromosome 4 or euchromatic dimethylation. In mammals, conversion of the H3K9 dimethyl- to the trimethyl-state by SETDB1 is strongly facilitated by the mAM cofactor [28]. Such a mechanism can also be envisaged for DmSETDB1, and *CG12340* is a candidate *Drosophila* homologue of mAM.

We could not detect any HMTase activity of DmSETDB1 in cell-free conditions. Immunopurified DmSETDB1, regardless of whether expressed in mammalian or in *Drosophila* S2 embryo cell lines, did not show any activity when tested on GST-H3, GST-H4, core histones, or oligonucleosomes, while mammalian SETDB1 produced under identical conditions showed robust H3 specific activity (unpublished data). We hypothesize that another protein or a post-translational modification is necessary for HMTase function of DmSETDB1. This activity would not be present in S2 cell line; this is consistent with the fact that overexpression of DmSETDB1 in S2 cells does not induce any increase in H3K9 mono-, di-, or trimethylation (unpublished data).

### DmSETDB1 Functionally Interacts with HP1

DmSETDB1 functions in association with HP1; HP1 is recruited when DmSETDB1^421–1,261^ is overexpressed and lost from some euchromatic bands and Chromosome 4 in the *DmSetdb1^10.1a^* mutant. In addition, HP1 is required for DmSETDB1-dependent repression of Chromosome 4 variegating transgenes [21,38–40]. We speculate that HP1 is recruited to chromatin by both the DmSETDB1 protein and the H3K9 methyl mark. Indeed, the DmSETDB1 protein is not able to recruit HP1, because the DmSETDB1^421–1,261^(H1195K) mutant protein does not influence HP1 localization. On the other hand, the H3K9 methyl mark alone is not sufficient to recruit HP1 [49]. Therefore, we hypothesize that HP1 recognizes the H3K9 methyl mark in association with DmSETDB1, or with another factor. The situation is similar for Suv39H1, where the protein itself does not recruit HP1, despite a direct interaction that is necessary for HP1 binding in collaboration with the H3K9 methyl mark [49]. We do not know if a direct DmSETDB1-HP1 interaction occurs, but two arguments in mammals argue in favor of this. First, KAP1 directly binds HP1 [50] and SETDB1 [27], and in such a complex, contacts between HP1 and SETDB1 are probable. Second, heterochromatin targeted HP1 recruits SETDB1 [51,52], although an intermediate factor cannot be excluded.

### DmSETDB1, SU(VAR)3–9, and Other Potential H3K9 HMTases

Although both DmSETDB1 and SU(VAR)3–9 methylate H3K9, one cannot substitute for the other. Indeed, in a mutant background for one enzyme, the other will not compensate for its absence. In addition, we can conclude that both enzymes function independently; SU(VAR)3–9-mediated H3K9 di- and trimethylation and HP1 deposition at the chromocenter are not affected in the *DmSetdb1^10.1a^* mutant context, and conversely, H3K9 mono- and dimethyl marks at euchromatic arms, dimethyl marks on Chromosome 4, and the associated HP1, are not affected in a *Su(var)3–9* mutant background [14,15]. Surprisingly, SU(VAR)3–9 is present on Chromosome 4; it is most probably recruited by HP1, but it does not induce any H3K9 methylation [14,15]. Thus, DmSETDB1 and SU(VAR)3–9 exert nonoverlapping and independent functions, suggesting that they accomplish distinct biological roles. We anticipate that at least one additional HMTase is involved in H3K9 methylation in *Drosophila*. H3K9 monomethylation at the chromocenter, H3K9 dimethylation at the telomeres, and some of the H3K9 mono- and dimethylation marks at euchromatic bands are not deposited by SU(VAR)3–9 nor DmSETDB1. One candidate, dG9a, was recently shown to methylate H3K9 and to localize to euchromatin [16,17].

### DmSETDB1 Is Required for Repression of Chromosome 4 Variegating Transgenes

The repressive function of DmSETDB1 demonstrated for Chromosome 4 is consistent with the fact that H3K9 methylation is generally found in association with transcriptional silencing [53,54]. Indeed, the mammalian SETDB1 homologue fulfills such a function [27,45,47,48]*.* DmSETDB1 could also be implicated positively in gene expression, since H3K9 di- and trimethylation, as well as HP1γ were recently found in the coding region of active genes [55,56]. One task will be to identify endogenous genes that are regulated by DmSETDB1 in euchromatin and at Chromosome 4. Genes located in the region 31 are potential candidates, given that the HP1 signal is lost in the *DmSetdb1^10.1a^* mutant. The second set of candidate genes are those physically associated with HP1 but not with SU(VAR)3–9. Greil et al. [57] performed large-scale mapping of HP1 and SU(VAR)3–9 targeted loci in embryonic Kc cells and showed that whereas HP1 and SU(VAR)3–9 bind together to transposable elements and pericentric genes, HP1 binds to many genes on Chromosome 4, mostly independently of SU(VAR)3–9. The latter, together with a class of euchromatic genes showing the same protein-factor occupation profile, possibly depend on DmSETDB1 for H3K9 methylation and regulation.

DmSETDB1 is the H3K9 HMTase responsible for heterochromatin silencing on Chromosome 4, because variegating transgenes are derepressed in a *DmSetdb1^10.1a^* mutant background. As both alleles have to be mutated in order to obtain an effect, the *DmSetdb1* gene is a recessive suppressor of variegation on Chromosome 4. Conversely, loss of a single dose of HP1 or SU(VAR)3–7 results in loss of silencing [21,38–40]. This difference could be explained by the fact that DmSETDB1 is an enzyme, whereas HP1 and SU(VAR)3–7 are dosage-sensitive structural components. Alternatively, DmSETDB1 might be present in excess. Heterochromatic variegating reporters are responding to an additional or missing dose of SU(VAR)3–9 when inserted on Chromosomes 2, 3, or X, but not on Chromosome 4 (mentioned in [18] as personal communication from K. Haynes [41]). This observation is henceforth explained by the fact that DmSETDB1 mediates H3K9 dimethylation on Chromosome 4. Conversely, and as expected, variegating expression responding to the SU(VAR)3–9 dosage is not under the control of DmSETDB1 (Figure 4F–4I). This corroborates once again that SU(VAR)3–9 and DmSETDB1 function independently. Mammalian SETDB1 is involved in epigenetic maintenance, since silencing is stably maintained for more than 40 population doublings, once it is established on an integrated reporter by a short transient pulse of the corepressor KAP1 that subsequently recruits SETDB1 and HP1 [58]. DmSETDB1 could also be involved in epigenetic maintenance; in that case, transient expression would suffice for long-term repression of Chromosome 4 variegating transgenes.

The arm of Chromosome 4 is composed of a minimum of three euchromatic domains interspersed with heterochromatic domains [21,38]. The variegating P elements that we tested were inserted within the banded region, in or at the edge of heterochromatic domains [38]. Chromosome 4 heterochromatic bands are qualitatively different from centromeric heterochromatin, as they are H3K9 dimethylated and regulated by DmSETDB1, not by SU(VAR)3–9. Two possibilities can be envisaged for the Chromosome 4 domains that are methylated by DmSETDB1. First, they could be representative of equivalent bands at euchromatic arms, which would be smaller and/or more dispersed, and therefore would not yet have been identified functionally. Alternatively, D. melanogaster Chromosome 4 could make use of specific machinery dedicated to gene regulation and/or epigenetic maintenance. The other well-known example of chromosome-specific regulation is the dosage compensation of sex chromosomes [59]. In that case, DmSETDB1 function would depend on partners or DNA sequences specific for Chromosome 4, such as for instance the Chromosome 4-specific factor POF [60,61], or the *Hoppel* element, also known as 1360, which is over-represented on the D. melanogaster Chromosome 4 [62], and which could be an initiation site for heterochromatin formation [21].

In conclusion we have characterized DmSETDB1 as a major nonheterochromatic H3K9 methyltransferase in *Drosophila*. We also demonstrated that *DmSetdb1* is an essential gene and that its loss has functional consequences on gene expression on Chromosome 4. This work represents an important step toward the understanding of the differential specificity and mode of action of distinct H3K9 HMTases and underlines a specific mode of regulation of Chromosome 4 in *Drosophila*.

## Materials and Methods

### *Drosophila* lines.

The 39C12, 39C72, 118E10, 118 E15, 6M193, 39C3, and 39C5 lines contain the P[*hsp26pt, hsp70-w*] element and are gifts from Sarah Elgin [21,22,40]. *Heidi* was described in [63]. The stocks *y w (v); P[ry^+^, 70FLP]4 P[v^+^, 70I-SceI]2B Sco/S^[2]^ CyO* and *w^1118^; P[ry^+^, 70FLP]10* were provided by Y. Rong and K. Golic. Description of other stocks can be found at FlyBase (http://flybase.bio.indiana.edu).

### Establishment of *DmSetdb1^421–1,261^* transgenic lines.

*DmSetdb1^421–1,261^* (CG30426) ORF was cloned by RT-PCR. *3HA- DmSetdb1^421–1,261^* carries in the N terminus a 3HA epitope derived from pBSKS-3HA [64]. *DmSetdb1^421–1,261^(H1195K)* point mutation was generated by PCR. All constructs were verified by sequencing. *3HA- DmSetdb1^421–1,261^* was cloned into pCaSpeR. *DmSetdb1^421–1,261^*, *DmSetdb1^421–1,261^(H1195K)*, *3HA- DmSetdb1^421–1,261^*, and *3HA- DmSetdb1^421–1,261^(H1195K)* were cloned into pUASP vector [65]. Cloning details are available upon request. Constructs were injected into *w^1118^* embryos with the pUChsπdelta2–3 plasmid at a 3:1 ratio. Transformant flies were selected with the *white* marker. DmSETDB1^421–1,261^ versions cloned in the pUASP vector and located on Chromosome 3 were recombined with the *daGal4* driver located on Chromosome 3. Homozygous *DmSetdb1^10.1a^* larvae were selected from the stock *w*; *Setdb^110.1a^ /CyO GFP*, where nonfluorescent homozygous mutant larvae were selected.

### Generation of *DmSetdb1* ^10.1a^ and *DmSetdb1* ^3HA^ alleles by homologous recombination.

*DmSetdb1^10.1a^* were generated as follows. Cloning 4.1-kb genomic DNA located 5′ from CG30426 as well as 3.9-kb located 3′ from CG30426, (corresponding respectively to positions 95154–91021 and 88189–84215 [NCBI]), were amplified with high fidelity Taq DNA polymerase (Roche, http://www.roche.com). PCR products were sequenced to ensure integrity of genes present in those regions. The 5′ amplified region was cloned into the NotI site of pW25 (a gift from K. Golic), and the 3′ region was cloned into the AscI site. The procedure for the targeting screen was performed as described previously [35,36]. Briefly the targeting construct was injected into the *w^1,118^* strain with the pUChsπdelta2–3 plasmid at a 3:1 ratio to obtain “donor” lines. A total of four independent donor lines on Chromosomes 3 or X were obtained. A total of 200 females of each donor line were crossed with *yw*; *70FLP*, *70I-SceI, Sco/CyO* males. We carried out two heat shocks on first- and second-instar larvae for one hour at 37 °C. From the progeny, 800 mosaic females carrying the *70FLP, 70 I-SceI* chromosome were crossed with *yw* homozygous males expressing *70FLP* constitutively. From the progeny, nonmosaic *white* positive flies were selected and further analyzed, to confirm that the *w^Hs^* marker replaced the coding region of *DmSetdb1*. The reduction step eliminates the *w^Hs^* marker flanked by two loxP sites. The homologous recombinants were crossed to the *yw;CyO P[w+ 70CreI/Sco*] (FlyBase) line expressing the Cre recombinase. From the progeny, *white* negative flies were further characterized, and deletion of *DmSetdb1* was confirmed by sequencing the region where homologous recombination occurred.

*DmSetdb1^3HA^* was generated using the following procedure. A 4.1-kb *Xba/Not* DNA fragment containing sequences 5′ of CG30426 (positions 95154–91021[NCBI]) with a *I-SceI* site inserted at position 93069 (EagI), a 3.0-kb *Xba/EagI* fragment containing CG30426 (positions 91020–88310), and a 3HA tag in at position 91020, were cloned into the pTV2 (NotI) vector [66]. In this clone, the ORF is conserved from CG30422 to CG30426, and the *I-CreI* site faces position 88312. All PCR products were sequenced. The targeting screen procedure is similar to the *DmSetdb1^10.1a^* allele. The reduction step involves a recombination that replaces the endogenous CG30426 with the 3HA-tagged CG30426 and deletes the *w^Hs^* marker. Females recombinant/SM5 were crossed with males *CyO/+;70-I Cre 1A/ TM3*. Heat shocks were made on first instar larvae 30 min at 37 °C, and variegated males were balanced with *w^1,118^; CyO;TM3/* T2-3Ap^Xa^ females. *w* ^−^ */CyO* flies were crossed with each other. Homozygote-reduced recombinant flies were analyzed by PCR. The region where the 3HA is inserted was sequenced.

### Western blot analysis.

Brains, salivary glands, and imaginal discs from third instar larvae were dissected in PBS, resuspended in 50 mM Tris (pH 7.8), 150 mM NaCl, 5 mM EDTA, 1% SDS, 1 mM PMSF, and protease inhibitors (Complete, Roche), boiled 10 min, and cleared by centrifugation. We separated 20-μg or 5-μg extract on 15% SDS-PAGE, and proteins were transferred on PVDF membrane (Millipore, http://www.millipore.com) by semi-dry transfer. Membranes were blocked in TBS, 0.1% tween, 5% non fat milk, hybridized in TBS, 0.1% tween, 1% non fat milk, with α-H3K9me1 (1/1000) (a gift from T. Jenuwein), α-H3K9me2 (1/1000) (a gift from T. Jenuwein), α-H3K9me3 (1/1000) (Upstate Biotechnology 07–523, http://www.upstate.com), α-H3 (1/5000) (Abcam 1791, http://www.abcam.com), α-HP1 (1/4000) (a gift from L. Wallrath), or α-α-tubulin (1/5000) (Sigma T 9026, http://www.sigmaaldrich.com). Membranes were washed with TBS, 0.1% tween, hybridized with HRP-coupled secondary antibody, washed, and revealed by chemoluminescence. Where indicated, the membranes were stripped and reprobed.

### Northern blot analysis.

Total RNA from 0–4-h and 0–18-h embryos, third instar larvae, and male and female adults was extracted using Trizol reagent (Invitrogen, http://www.invitrogen.com). We separated 20 μg RNA from each sample on 1% agarose-formaldehyde gel, transferred to Hybond-N+ membrane (Amersham, http://www.amersham.com), and UV crosslinked. Membrane was hybridized in Rapid-Hyb buffer (Amersham) with a probe covering nucleotides 2,399–3,789 of *DmSetdb1* ORF and subsequently with an RNA-loading control probe recognizing *rp49*. Probes were radioactively ^32^P labeled using the Redi-prime labeling kit (Amersham) as described by the manufacturer.

### Immunostaining of polytene chromosomes.

Polytene chromosomes were performed as described previously [67]. Briefly, salivary glands were dissected in Cohen's buffer, fixed for 2 min in 2% formaldehyde, 2% Triton X-100, and then squashed in 2% formaldehyde, 45% acetic acid. Slides were hybridized with the following primary antibodies: α-HA (1/200) (Covance MMS-101R, http://www.covance.com), α-HP1 (1/400) (a gift from L. Wallrath), α-H3K9me1 (1/200) (a gift from T. Jenuwein), α-H3K9me2 (1/100) (Upstate Biotechnology 07–441), and α-H3K9me3 (1/200) (Upstate Biotechnology 07–523).

### Suppression of Position Effect Variegation in *DmSetdb1* mutant context.

Picture of flies at pupal stage with the following genotype were taken: Figure 4A: *w/w; DmSetdb1^10.1a^/+; 39C12/+* and *w/w; DmSetdb1^10.1a^/ DmSetdb1^10.1a^; 39C12/+*. Figure 4B: *w/w; DmSetdb1^10.1a^/+; 39C72/+* and *w/w; DmSetdb1^10.1a^/DmSetdb1^10.1a^; 39C72/+.* Figure 4C: *w/w; DmSetdb1^10.1a^/+; 118E10/+* and *w/w; DmSetdb1^10.1a^/DmSetdb1^10.1a^; 118E10/+*. Figure 4D: *w/w; DmSetdb1^10.1a^/+; 6M193/+* and *w/w; DmSetdb1^10.1a^/DmSetdb1^10.1a^; 6M193/+*. Figure 4E: *w/w; DmSetdb1^10.1a^/+; 118E15/+* and *w/w; DmSetdb1^10.1a^/DmSetdb1^10.1a^; 118E15/+.* Figure 4F: *w/w; DmSetdb1^10.1a^/39C3* and *w/w; DmSetdb1^10.1a^ 39C3/ DmSetdb1^10.1a^*. Figure 4G: *In(1)w^m4h^ /w; DmSetdb1^10.1a^/+* and *In(1)w^m4h^/w; DmSetdb1^10.1a^/ DmSetdb1^10.1a^*. Figure 4H: *w/w; DmSetdb1^10.1a^/Heidi* and *w/w; DmSetdb1^10.1a^ Heidi/DmSetdb1^10.1a^*. Figure 4I: *w/w; DmSetdb1^10.1a^/39C5* and *w/w; DmSetdb1^10.1a^ 39C5/ DmSetdb1^10.1a^*. Lines *39C12*, *39C72*, *118E10*, *118E15,* and *6M193* [22] are on Chromosome 4, and lines *39C3, 39C5* [22], and *Heidi* [63] are on Chromosome 2.

## Supporting Information

Figure S1 Structure of Polytene Chromosomes of Third Instar Larvae Structure of polytene chromosomes of (A) wild-type, (B) *DmSetdb1^10.1a^* homozygous mutant (*DmSetdb1^10^* ^.1a^/*DmSetdb1^10^* ^.1a^), and (C and D) overexpressing DmSETDB1^421–1,261^ (*UAS-DmSetdb1^421–1,261^ daGal4* homozygotes) of third instar larvae are shown. Polytene chromosomes were stained with orcein. “C” points at the chromocenter and “4” at Chromosome 4. Overexpression of DmSETDB1^421–1,261^ causes loosening of the chromocenter and constrictions on the arms. The polytene chromosomes of *DmSetdb1^10.1a^* homozygous mutant do not present any abnormalities.
(1.9 MB PPT)

### Accession Numbers

The Flybase (http://www.flybase.org) accession numbers for the *Drosophila DmSetdb1* gene are *CG30422* and *CG30426*.

The National Center for Biotechnology Information (NCBI) (http://www.ncbi.nlm.nih.gov) accession number for *DmSetdb1* full insert cDNA is BT023947. The NCBI accession number for 4.1-kb genomic DNA located 5′ from *CG30426* as well as 3.9-kb located 3′ from *CG30426* (positions 95154–91021 and 88189–84215, respectively) is AE003465.

## Abbreviations

H3K9: histone H3 lysine 9
HMTase: histone methyltransferase
HP1: heterochromatin protein 1
K: lysine
MBD: methyl CpG binding
ORF: open reading frame
